# Spinal Stenosis and Carpal Tunnel Syndrome as Surrogates of Transthyretin Amyloid Cardiomyopathy

**DOI:** 10.1016/j.jacadv.2025.102545

**Published:** 2026-01-20

**Authors:** Laura De Michieli, Susan Geyer, Ellen McPhail, Mohamad Bydon, Benjamin D. Elder, Julie L. Rosenthal, Mary Jurisson, Sanjeev Kakar, Alberto Cipriani, Omar AbouEzzeddine, Surendra Dasari, Shaji Kumar, Morie Gertz, Martha Grogan, Angela Dispenzieri

**Affiliations:** a Department of Cardiovascular Medicine, Mayo Clinic and Medical School, Rochester, Minnesota, USA; b Department of Cardiac, Thoracic and Vascular Sciences and Public Health, University of Padua, Padua, Italy; c Department of Quantitative Health Sciences, Mayo Clinic, Rochester, Minnesota, USA; d Department of Laboratory Medicine and Pathology, Mayo Clinic, Rochester, Minnesota, USA; e Department of Neurological Surgery, Mayo Clinic, Rochester, Minnesota, USA; f Department of Cardiovascular Medicine, Mayo Clinic, Phoenix, Arizona, USA; g Department of Physical Medicine and Rehabilitation, Mayo Clinic, Rochester, Minnesota, USA; h Division of Hand and Upper Extremity Surgery, Department of Orthopedics, Mayo Clinic, Rochester, Minnesota, USA; i Department of Qualitative Health Sciences, Mayo Clinic, Rochester, Minnesota, USA; j Division of Hematology, Mayo Clinic, Rochester, Minnesota, USA

**Keywords:** amyloidosis, carpal tunnel syndrome, heart failure, lumbar spinal stenosis, overall survival, population study

## Abstract

**Background:**

Lumbar spinal stenosis (LSS) and carpal tunnel syndrome (CTS) often predate transthyretin amyloid cardiomyopathy.

**Objective:**

The aim of this study was to determine if patients with LSS/CTS have higher rates of cardiovascular events and worse survival, even in the absence of recognized transthyretin amyloid cardiomyopathy and the incidence of amyloidosis in patients with LSS/CTS.

**Methods:**

Retrospective population-based cohort study of patients (cases) with LSS/CTS diagnosis between 1995 and 2015 (50-90 years old) identified through Rochester Epidemiology Project (Minnesota, USA). Cases were matched with 3 age- and sex-matched controls. Outcomes—investigated with comprehensive regression models—were heart failure, atrial arrhythmias, device implantation, composite endpoint, survival, and incident amyloidosis.

**Results:**

A total of 6,076 LSS cases and 16,205 controls and 6,664 CTS cases and 17,462 controls were included. Compared to controls, LSS and CTS cases had more comorbidities at baseline and higher likelihood of cardiac events. Survival was worse in LSS cases vs controls (HR: 1.26; 95% CI: 1.19-1.33; *P* < 0.001) but similar in CTS cases vs controls (HR: 0.95; 95% CI: 0.90-1.01; *P* = 0.10). For LSS and CTS groups, respectively, the 10-year rate of amyloidosis diagnosis was 1.1% (vs 0.5% for controls, cause-specific HR: 2.18; *P* < 0.001) and 0.9% (vs 0.3%, cause-specific HR: 2.87; *P* < 0.001). Patients with both LSS and CTS had a 10-year incidence rate of amyloidosis of 1.45%.

**Conclusion:**

LSS and CTS were associated with increased likelihood of cardiovascular events and amyloidosis diagnosis. This highlight the importance of a dedicated assessment of cardiovascular symptoms for LSS/CTS patients, particularly if both conditions are present.

Cardiac amyloidosis (CA) is now a well-recognized cause of heart failure (HF).^1^^,^^2^ Early diagnosis of transthyretin amyloid (ATTR) cardiomyopathy (ATTR-CM), both wild type and hereditary, is essential because of the availability of treatments that can increase survival and improve quality of life.^3^, ^4^, ^5^, ^6^ Lumbar spinal stenosis (LSS) and carpal tunnel syndrome (CTS) are frequently associated with ATTR-CM, presenting years before cardiac diagnosis.^1^ However, the association of LSS and CTS with future development of ATTR-CM in the general population is yet to be defined, challenging routine pathologic evaluation of these specimens for ATTR infiltration.

Histopathological studies have reported a high frequency of ATTR amyloid deposits in ligamentous flavum specimens after LSS surgery, particularly in older patients;^7^^,^^8^ however, the minority of patients are diagnosed with ATTR-CM^9^, ^10^, ^11^ at the time of surgery, presumably due to the lag time between amyloid deposition in soft tissues vs myocardium. A prospective follow-up study recently reported that around 15% of subjects with high-degree ATTR deposition in the ligamentous flavum were diagnosed with wild-type ATTR-CM (ATTRwt-CM) 6 years after surgery.^12^ CTS is present in approximately 50% of referred patients with ATTR-CM, and symptoms of CTS precede the diagnosis by 5 to 10 years.^1^ Among patients who underwent bilateral CTS release in the preceding 5 to 15 years prospectively screened for ATTR-CM, 5% of patients had early-stage ATTR-CM, rising to 20% in nonobese men >70 years.^13^

Some have proposed that evaluation of ligamentum flavum and flexor tenosynovium specimens at the time of surgery could provide an opportunity for early diagnosis of ATTR-CM;^9^^,^^13^^,^^14^ however, rigorous data to support such practice are lacking. We hypothesized that if LSS and CTS were prodromal conditions for ATTR-CM, these patients would manifest a higher rate of cardiovascular (CV) events and worse survival. Moreover, they would manifest a higher rate of incident amyloidosis diagnoses. If true, histological evaluation of ligamentum flavum/flexor tenosynovium specimens at surgery and/or a dedicated cardiology follow-up of selected patients might be advisable as a means of earlier diagnosis for ATTR-CM or, more rarely, light chain amyloidosis.

## Methods

### Study design and data collection

This study was approved by the Mayo Clinic and Olmsted Medical Center Institutional Review Boards and informed consent was waived due to the study design. We performed a retrospective cohort study of patients between 50 and 90 years old with a history of LSS and/or CTS documented between 1995 and 2015, identified among residents of Olmsted County (Minnesota, USA). Patients were identified through the Rochester Epidemiology Project (REP),^15^, ^16^, ^17^ a medical record system linking together all the medical records of Olmsted County residents from multiple health care providers and institutions.

Patients with a positive history for LSS and/or CTS were identified based on International Classification of Diseases (ICD) codes (Supplemental Table 1). Detailed study flowcharts for the 2 cohorts are reported in Supplemental Figure 1. Index date was defined as the one in which LSS/CTS diagnosis or procedure code was used for the first time. Three age- and sex-matched controls without any reported code of LSS/CTS and seen during the same timeframe were randomly selected from the same population and matched without replacement. We analyzed patients diagnosed with LSS and those with CTS (and their respective controls) first as 2 separate cohorts, also differentiating between LSS/CTS surgery and diagnosis codes. We also evaluated the subset of patients with codes for both LSS and CTS. Baseline characteristics and comorbidities of both case-control cohorts were obtained through the REP registry. Baseline comorbidities were defined as such if coded in the charts before or up to 90 days after the index date. Patients with a pre-existing diagnosis of amyloidosis were excluded (Supplemental Table 2).

### Study endpoints

Due to the underdiagnosis of ATTR-CM in the study period, we used as clinical endpoints potential overt manifestations of this condition and specific ICD codes for amyloidosis. Through ICD codes (both inpatient and outpatient), we investigated the occurrence of cardiac events of interest (CEI), including congestive HF (CHF), atrial fibrillation/flutter (AF/AFL), and pacemaker (PM)/implantable cardiac defibrillator (ICD) implantation, individually and as a composite endpoint. These endpoints were chosen accordingly to existing literature^18^ and because they represent the most frequent complications potentially related to ATTR-CM.^19^ A subsequent CEI was defined as an event that occurred at least 90 days after the index date. For each of the CEI, the primary analyses were conducted on patients without pre-existing CEI. Similar analyses were conducted including patients with pre-existing CEI (except for PM/ICD implantation), but they are relegated to supplemental figures/tables. We also assessed the incidence of amyloidosis diagnosis using ICD codes (Supplemental Table 2); considering the potential difficulties in using ICD codes in this setting, we included all amyloidosis-related codes listed in Supplemental Table 2. We did not perform further clinical or histological confirmation, but we relied on the diagnoses coded by the treating physicians. Finally, survival was evaluated in LSS/CTS cohorts and compared between cases and controls.

### Statistical analysis

Conditional logistic regression models were used to evaluate baseline prevalence of comorbidities between cases vs controls. CEI (HF, AF/AFL, and PM/ICD) were summarized by group. When cumulative incidence of CEI was evaluated using competing risk regression models for stratified data, death was treated as a competing risk, and the models accommodated the correlated nature of the data with cases and matched controls. We reported cause-specific HRs (csHR) estimated from the competing risk models based on Fine and Gray^20^ methods, and proportionality of subdistribution hazards was assessed for all endpoints. Survival was defined as the time from index date to time of death. For all analyses, follow-up was extended up to 2018, and patients were followed until death, censoring, or end of the study period, whichever came first. Survival between cases vs controls was compared using frailty expectation-maximization algorithms for each cohort, where these models accommodate the correlated nature of time to event outcomes with the matched case-control design. Multivariable analyses were conducted to assess case-control differences while adjusting for confounders of interest, namely relevant comorbidities such as malignancy, hyperlipidemia, diabetes, cerebrovascular disease, chronic obstructive pulmonary disease, coronary artery disease, chronic kidney disease, peripheral vascular disease, systemic hypertension, and prior myocardial infarction. A directed acyclic graph is reported in the Supplemental Methods. Statistical analyses were conducted using the R statistical program (R Foundation, v4.2.2).

## Results

### Cohort characteristics

A total of 6,076 cases were included in the LSS cohort along with 16,205 controls matched on biological sex and age (Table 1). Of the cases, 55% were females, mean age 66.5 years, and 1,011 (16.6%) carried both a diagnosis code for LSS and a procedure code for LSS surgery. LSS cases had a significantly higher baseline prevalence of CV and non-CV comorbidities, including prior CHF, AF/AFL, PM/ICD implantation, and the composite of CHF, AF/AFL, and PM/ICD.

**Table 1:** Baseline Characteristics of LSS and CTS Cases and Their Matched Controls

	LSS Cohort			CTS Cohort		
	Controls (n = 16,205)	LSS Cases (n = 6,076)	OR^a^ (95% CI)	Controls (n = 17,462)	CTS Cases (n = 6,664)	OR^a^ (95% CI)
Female, n (%)	8,900 (55)	3,345 (55)		11,263 (64.5)	4,330 (65.0)	
Age at index date, y, mean (SD)	65.8 (11.7)	68.3 (10.9)		60.8 (11.7)	63.1 (10.7)	
Types of codes reported						
Diagnostic only, n (%)		5,065 (83.4%)			4,468 (67.0)	
Procedure + diagnostic, n (%)		1,011 (16.6%)			2,196 (33.0)	
Prior history of:						
Heart failure, n (%)	1,114 (6.9)	648 (10.7)	1.69 (1.52-1.88)	771 (4.4)	399 (6.0)	1.47 (1.29-1.67)
AF/AFL, n (%)	1,331 (8.2)	846 (13.9)	1.90 (1.73-2.10)	3,193 (18.3)	1,772 (26.6)	1.72 (1.60-1.85)
PM/ICD, n (%)	408 (2.5)	210 (3.5)	1.40 (1.17-1.66)	256 (1.5)	147 (2.2)	1.54 (1.25-1.90)
HF, AF/AFL, and/or PM/ICD, n (%)	2,026 (12.5)	1,215 (20.0)	1.87 (1.72-2.03)	3,460 (19.8)	1,905 (28.6)	1.74 (1.62-1.87)
Systemic hypertension, n (%)	7,934 (49.0)	4,177 (68.7)	2.55 (2.39-2.73)	6,766 (38.7)	3,666 (55.0)	2.17 (2.03-2.31)
Diabetes mellitus, n (%)	4,786 (29.5)	2,641 (43.5)	1.95 (1.83-2.08)	3,979 (22.8)	2,402 (36.0)	2.05 (1.92-2.19)
Hyperlipidemia, n (%)	9,079 (56.0)	4,381 (72.1)	2.15 (2.01-2.30)	8,120 (46.5)	4,265 (64.0)	2.21 (2.07-2.35)
Coronary artery disease, n (%)	3,074 (19.0)	1,873 (30.8)	2.10 (1.95-2.26)	2,172 (12.4)	1,374 (20.6)	2.02 (1.87-2.19)
Previous MI, n (%)	1,019 (6.3)	570 (9.4)	1.59 (1.42-1.78)	694 (4.0)	390 (5.9)	1.56 (1.37-1.78)
Cerebrovascular disease, n (%)	1,798 (11.1)	1,216 (20.0)	2.10 (1.93-2.28)	1,289 (7.4)	732 (11.0)	1.63 (1.47-1.80)
Peripheral vascular disease, n (%)	2,344 (14.5)	1,816 (29.9)	2.84 (2.63-3.07)	1,623 (9.3)	1,099 (16.5)	2.06 (1.89-2.25)
Chronic kidney disease, n (%)	1,087 (6.7)	713 (11.7)	1.91 (1.72-2.12)	722 (4.1)	489 (7.3)	1.91 (1.69-2.17)
COPD, n (%)	1,519 (9.4)	909 (15.0)	1.73 (1.59-1.90)	1,145 (6.6)	653 (9.8)	1.58 (1.43-1.75)
Malignancy (any type excluding skin tumors), n (%)	3,827 (23.6)	2,026 (33.3)	1.69 (1.58-1.81)	3,099 (17.7)	1,521 (22.8)	1.43 (1.33-1.53)
Number of comorbidities at baseline^b^, mean (SD)	2.25 (2.04)	3.35 (2.14)	1.37 (1.35-1.39)	1.70 (1.79)	2.49 (1.97)	1.34 (1.32-1.37)

AF/AFL = atrial fibrillation/flutter; COPD = chronic obstructive pulmonary disease; CTS = carpal tunnel syndrome; ICD = implantable cardiac defibrillator; HF = heart failure; LSS = lumbar spinal stenosis; MI = myocardial infarction; PM = pacemaker.

a Results from conditional logistic regression model accounting for matched sets of cases and age- and sex-matched controls. Comparison across all groups is significantly different at a *P* value of <0.001.

b Comorbidities at baseline (ie, before and up to 90 days after the index date), not including HF, AF/AFL, and/or PM/ICD.

A total of 6,664 cases were included in the CTS cohort along with 17,462 matched controls (Table 1). Most of the CTS cases were females (65%), mean age 61.4 years; 4,468 patients (67%) only had a diagnosis CTS code, 2,196 (33%) also had a procedure code for CTS surgery. CTS cases consistently had a significantly higher prevalence of CV and non-CV comorbidities, including prior CHF, AF/AFL, PM/ICD implantation, and the composite of CHF, AF/AFL, and PM/ICD implantation.

The 1,181 patients with a history of both LSS and CTS (Supplemental Table 3) tended to be more similar to LSS only cases in terms of comorbidities.

### Incidence of CEI

#### LSS cohort

**Figure 1:**
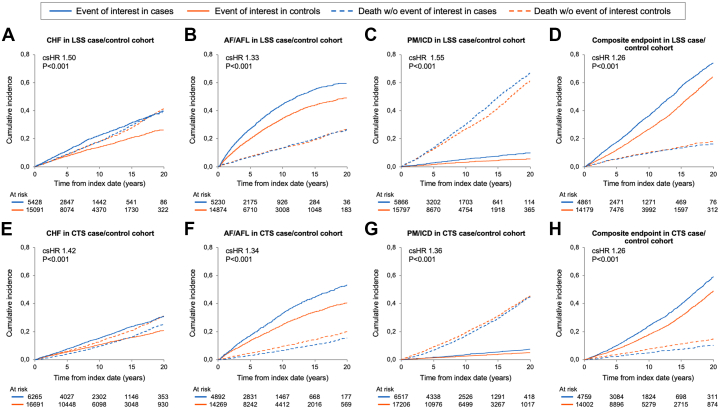
**Cumulative Incidence of Cardiac Events of Interest** Cumulative incidence of heart failure (HF), atrial fibrillation/flutter (AF/AFL), pacemaker and implantable cardioverter defibrillator (PM/ICD) implantation, and of the composite outcome, including death as a competing risk, in patients with lumbar spinal stenosis (LSS) (A-D) and carpal tunnel syndrome (CTS) (E-H) without previous history of these events and matched controls. (A) HF in LSS cohort, (B) AF/AFL in LSS cohort, (C) PM/ICD in LSS cohort; and (D) composite outcome (HF, AF/AFL, or PM/ICD) in LSS cohort. (E) HF in CTS cohort; (F) AF/AFL in CTS cohort, (G) PM/ICD in CTS cohort, and (H) composite outcome (HF, AF/AFL, or PM/ICD) in CTS cohort. CHF = congestive heart failure; csHR = cause-specific HR.

**Figure 2:**
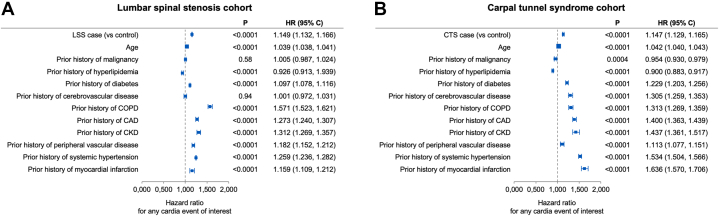
**Multivariable Competing Risk Regression Model for Any Cardiac Event** Forest plot showing multivariable competing risk regression model for any cardiac event of interest (HF, AF/AFL, or PM/ICD implantation) in patients with (A) lumbar spinal stenosis (LSS) and (B) carpal tunnel syndrome (CTS) and without a prior history of the cardiac events of interest. CAD = coronary artery disease; COPD = chronic obstructive pulmonary disease; CKD = chronic kidney disease; other abbreviations as in Figure 1.

With a median follow-up of 8.8 years from the index date (95% CI: 8.6-9.1 years), cardiac composite endpoint occurred in 8,335 (37.4%) patients, of whom 5,942 did not have a prior history of the CEI. The respective estimated 10-year cumulative incidence rates of CHF, AF/AFL, PM/ICD, and composite outcome among cases without prior CEI were significantly higher than controls (Figures 1A to 1D, Table 2, Central Illustration): CHF, 22.5% in cases vs 14.0% in controls; AF/AFL, 44.3% in cases vs 34.5% in controls; PM/ICD, 5.5% in cases vs 3.3% in controls; and composite outcome, 36.3% in cases vs 26.9% in controls, all *P* < 0.001. The forest plot (Figure 2) demonstrates the multivariate csHR and corresponding differential hazards of developing any of the CEI and reveals that the risk conferred by LSS is independent of and approaches that of other relevant comorbidities such as prior coronary artery disease, prior myocardial infarction, and chronic kidney disease. As shown in Table 2 and Supplemental Figure 2, LSS was a risk marker for incident CEI regardless of whether there was a surgical procedure or not.

**Table 2:** LSS and CTS Cases Versus Controls: Competing Risk Regression Models for Cardiac Events of Interest

Models	Cardiac Event of Interest			
	CHF	AF/AFL	PM/ICD	Any Cardiac Event of Interest^a^
LSS cohort				
M1^b^: LSS case (vs control) csHR (95% CI)	1.50 (1.47-1.53)	1.33 (1.31-1.35)	1.55 (1.50-1.61)	1.26 (1.25-1.28)
10-year rate (SD)				
LSS cases	22.5% (0.7%)	44.3% (0.9%)	5.5% (0.4%)	36.3% (0.9%)
Controls	14.0% (0.4%)	34.5% (0.5%)	3.3% (0.2%)	26.9% (0.5%)
M2^b^: Controls csHR (95% CI)	Reference	Reference	Reference	Reference
10-year rate (SD)	14.0% (0.4%)	34.5% (0.5%)	3.3% (0.2%)	26.9% (0.5%)
LSS case with diagnosis (no procedure)				
csHR (95% CI)	1.55 (1.51-1.58)	1.31 (1.29-1.33)	1.56 (1.50-1.62)	1.28 (1.26-1.30)
10-year rate (SD)	23.1% (0.8%)	43.7% (1.0%)	5.5% (0.4%)	36.8% (1.0%)
LSS case with procedure				
csHR (95% CI)	1.32 (1.25-1.39)	1.40 (1.35-1.45)	1.53 (1.44-1.62)	1.20 (1.15-1.24)
10-year rate (SD)	19.8% (1.5%)	46.8% (2.0%)	5.2% (0.8%)	34.5% (1.9%)
CTS cohort				
M3^b^: CTS case (vs control) csHR (95% CI)	1.42 (1.40-1.45)	1.34 (1.32-1.36)	1.36 (1.31-1.40)	1.26 (1.24-1.28)
10-year rate (SD)				
CTS cases	15.4% (0.6%)	33.0% (0.8%)	3.6% (0.3%)	24.3% (0.8%)
Controls	10.6% (0.3%)	24.9% (0.4%)	2.5% (0.1%)	17.9% (0.4%)
M4^b^: Controls csHR (95% CI)	Reference	Reference	Reference	Reference
10-year rate (SD)	10.6% (0.3%)	24.9% (0.4%)	2.5% (0.3%)	17.9% (0.4%)
CTS case with diagnosis (no procedure)				
csHR (95% CI)	1.44 (1.41-1.48)	1.35 (1.32-1.37)	1.28 (1.23-1.33)	1.29 (1.27-1.32)
10-year rate (SD)	15.5% (0.7%)	31.8% (1.0%)	3.6% (0.4%)	24.6% (0.9%)
CTS case with procedure				
csHR (95% CI)	1.39 (1.39-1.43)	1.33 (1.29-1.37)	1.50 (1.41-1.59)	1.21 (1.17-1.24)
10-year rate (SD)	15.0% (0.9%)	35.1% (1.4%)	3.6% (0.5%)	23.6% (1.3%)

Table values reflect the cause-specific HR (csHR) of the case in relation to the age- and sex-matched controls. Cumulative incidences are calculated with death as a competing risk and only patients who did not have a prior history of the cardiac event of interest are included.

CHF = congestive heart failure; other abbreviations as in Table 1.

a CHF, AF/AFL, and/or PM/ICD implantation.

b *P* < 0.001.

Findings were similar even if patients with pre-existing CEI were included in the analyses (all *P* < 0.001) (Supplemental Figure 3, Supplemental Table 4). Again, on multivariable analysis adjusting for relevant CV and non-CV comorbidities (Supplemental Figure 4), the independent role of LSS as a risk marker for any CEI was confirmed. Differences in the event rates based on biological sex is reported in Supplemental Table 5. CEI were generally more frequent in men compared to women in both cases and controls with the exceptions of CHF and the composite outcome among cases, for whom frequency was no different for men and women.

#### CTS cohort

With a median follow-up of 10.4 years (95% CI: 10.1-10.6 years), 8,111 (33.6%) patients experienced the composite endpoint, of whom 5,107 did not have a prior history of the CEI. The respective estimated 10-year cumulative incidence rate of CHF, AF/AFL, PM/ICD, and composite outcome was significantly higher in CTS cases than controls (Figures 1E to 1H, Table 2, Central Illustration): CHF, 15.4% in cases vs 10.6% in controls; AF/AFL, 33.0% in cases vs 24.9% in controls; PM/ICD, 3.6% in cases vs 2.5% in controls; and composite outcome, 24.3% in cases vs 17.9% in controls, all *P* < 0.001. The forest plot (Figure 2B) demonstrates the multivariate csHR and corresponding differential hazards of developing any of the CEI and reveals that the risk conferred by CTS is independent of and approaches that of other relevant CV comorbidities. As shown in Table 2 and Supplemental Figure 2, CTS was a risk marker for incident CEI regardless of whether there was a surgical procedure or not.

Findings were similar if all cases were included, even those with pre-existing CEI (all *P* < 0.001) (Supplemental Figure 3, Supplemental Table 4). Again, on multivariable analysis adjusting for relevant CV and non-CV comorbidities (Supplemental Figure 4), the independent role of CTS as a risk marker for CEI was confirmed. Differences in the event rates based on biological sex is reported in Supplemental Table 5. CEI were generally more frequent in men compared to women.

#### Both LSS and CTS cohort

For the 1,181 patients with both LSS and CTS, hazards of manifesting CHF (csHR: 1.95; 95% CI: 1.71-2.22; *P* < 0.0001), AF/AFL (csHR: 1.52; 95% CI: 1.36-1.70; *P* < 0.0001), PM/ICD implantation (csHR: 1.72; 95% CI: 1.33-2.23; *P* < 0.0001), and the composite outcome (csHR: 1.61; 95% CI: 1.44-1.81; *P* < 0.0001) was higher compared to their matched controls (Supplemental Table 6, Supplemental Figure 5).

### Survival during long-term follow-up

**Figure 3:**
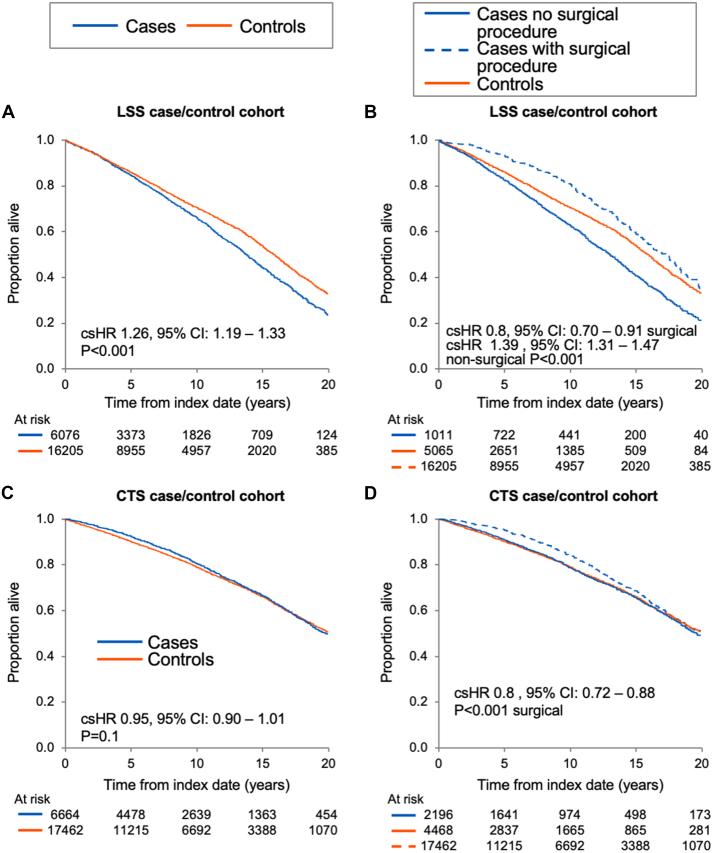
**Survival in Lumbar Spinal Stenosis and Carpal Tunnel Syndrome Patients** (A) Lumbar spinal stenosis (LSS) cases vs controls. (B) LSS cases with only a diagnosis vs LSS cases who have a reported procedure vs matched controls. (C) Carpal tunnel syndrome (CTS) cases vs controls. (D) CTS cases with only a diagnosis vs CTS cases who have a reported procedure vs matched controls. Abbreviations as in Figure 1.

For the LSS cohort, 6,782 deaths were observed, and survival was significantly worse in LSS cases than matched controls (HR: 1.26; 95% CI: 1.19-1.33; *P* < 0.001) (Figure 3A); however, LSS cases with a procedure code had a better survival (HR: 0.80; 95% CI: 0.70-0.91; *P* < 0.0001), whereas those without a procedure code had significantly worse survival (HR: 1.39; 95% CI: 1.31-1.47; *P* < 0.001) (Figure 3B) compared to the respective matched controls.

For the CTS cohort, 5,836 deaths were reported overall. Survival was similar between CTS cases and controls (HR: 0.95; 95% CI: 0.90-1.01; *P* = 0.10) (Figure 3C), although patients who had a CTS surgery code had superior survival vs the controls (HR: 0.80; 95% CI: 0.72-0.88; *P* < 0.001) (Figure 3D).

For the LSS-CTS case-control cohort, 1,425 deaths were reported. Cases with both LSS and CTS diagnoses had a lower hazard of death (HR: 0.82, 95% CI: 0.74-0.90; *P* < 0.0001) compared to their matched controls, but when the analysis was restricted to those who had a diagnosis of LSS and CTS within the same year, survival was worse for the cases (HR: 1.42; 95% CI: 1.12-1.81; *P* = 0.004).

### Incidence of amyloidosis diagnosis

#### LSS cohort

During follow-up, 131 patients (58 LSS cases and 73 controls) had an incident diagnosis of amyloidosis. LSS cases had a significantly higher probability of incident amyloidosis diagnosis (csHR: 2.18; 95% CI: 2.10-2.26; *P* < 0.001) than matched controls (Supplemental Figure 6A) with an estimated 10-year rate of an amyloidosis diagnosis of 1.1% vs 0.5% for LSS cases vs controls.

When considering the incidence of amyloidosis diagnoses by biological sex, the estimated 10-year amyloidosis incidence rate was 0.5% in women vs 1.2% in men (*P* = 0.0001) overall. Among LSS cases, the 10-year rates of amyloidosis diagnoses for women and men were 0.7% and 2.1%, respectively, *P* = 0.0001. In the matched controls, there was a tendency toward higher rate in men, with a 10-year estimated amyloidosis rate of 0.4% in women vs 0.8% in men (*P* = 0.062).

#### CTS cohort

A total of 118 patients had an incident diagnosis of amyloidosis (62 CTS cases and 56 controls). CTS cases had a significantly higher probability of incident amyloidosis diagnosis (csHR: 2.87; 95% CI: 2.70-3.05; *P* < 0.001) than matched controls (Supplemental Figure 6B) with an estimated 10-year rate of an amyloidosis diagnosis of 0.9% vs 0.3% for CTS cases vs controls. When considering the diagnostic rate by biological sex, in the overall CTS cohort, the estimated 10-year rates of amyloidosis diagnoses were 0.4% for women vs0.8% for men (*P* = 0.0004) and this difference was consistent in CTS cases (0.7% vs 1.4%, *P* = 0.003) and controls (0.3% vs 0.5%, *P* = 0.044).

#### Both LSS and CTS cohort

**Figure 4:**
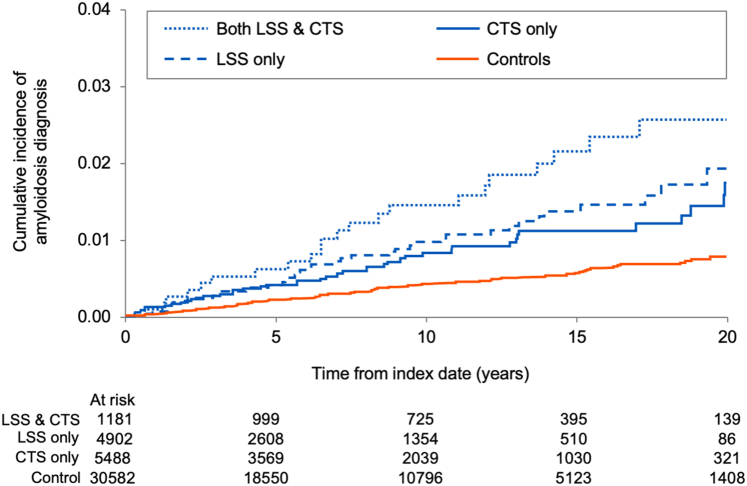
**Incidence of Amyloidosis Diagnoses Across Different Cohorts** Cumulative incidence of amyloidosis across both cohorts and in patients with lumbar spinal stenosis (LSS) and carpal tunnel syndrome (CTS) vs matched controls. Death without amyloidosis is treated as a competing risk. Abbreviations as in Figure 1.

Patients with both an LSS and CTS diagnosis code had a higher incidence rate of amyloidosis (Figure 4), with a 10-year incidence rate of 1.45% vs estimated rates of 0.97% and 0.83% for those with only LSS diagnosis or CTS diagnosis codes, respectively.

**Central Illustration:**
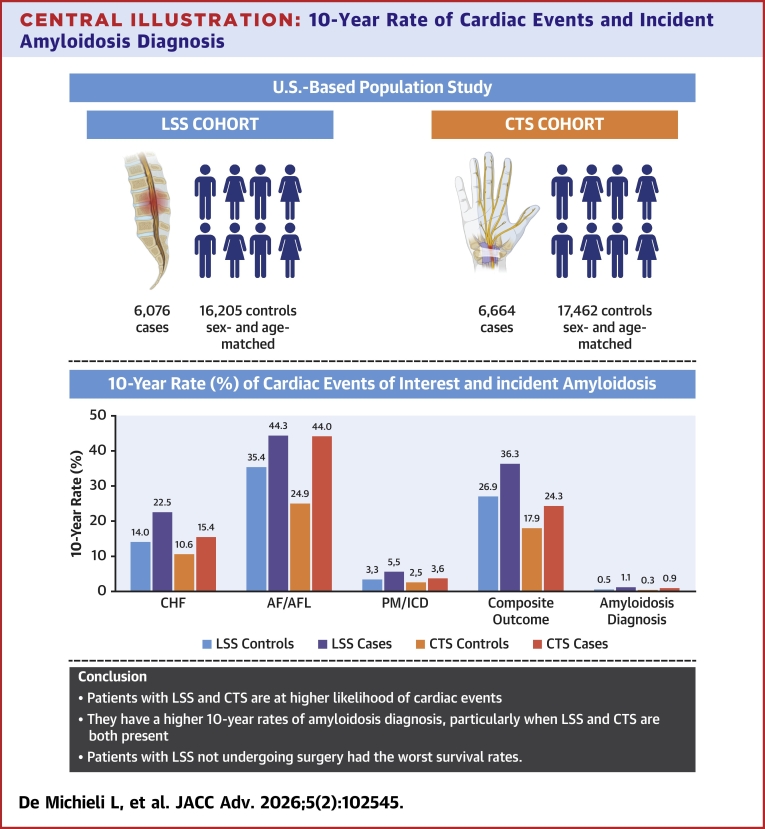
**10-Year Rate of Cardiac Events and Incident Amyloidosis Diagnosis** Ten-year rates of the cardiac events of interest and of incident amyloidosis diagnoses in lumbar spinal stenosis (LSS) cases and their matched controls (light blue and blue, respectively) and carpal tunnel syndrome (CTS) cases and their matched controls (light orange and orange, respectively). Comparisons between cases and matched controls were all *P* < 0.001. AF/AFL = atrial fibrillation/flutter; CHF = congestive heart failure; CTS = carpal tunnel syndrome; LSS = lumbar spinal stenosis; PM/ICD = pacemaker/implantable cardioverter defibrillator.

## Discussion

This is a retrospective population-based study designed to investigate the potential relationship between LSS and CTS and future CEI, which we assumed might be a surrogate for undiagnosed ATTR-CM. We present several important findings. First, compared to matched controls, patients with LSS or CTS were at a higher probability of CEI, including HF, AF/AFL, and PM/ICD implantation, all potential manifestations of undiagnosed ATTR-CM, even when correcting for comorbidities and competing risk of death. Second, LSS-only and CTS-only patients also were at a higher probability of subsequent incident amyloidosis diagnoses. Third, patients with both LSS and CTS had the highest probability of cardiac events and of developing amyloidosis compared to those with a single diagnosis. Finally, patients with LSS had inferior survival compared to matched controls, although this was not the case for patients with CTS. These data confirm the association between ligamentous disease and future CV events possibly related to ATTR-CM, highlighting its role as a clinical marker of potentially undiagnosed amyloid-related disease. Nevertheless, due to the retrospective nature and timeframe of the study, these findings likely underestimate the actual “penetrance” of ligamentous disease and future amyloid diagnoses.

We observed that patients with LSS or CTS have a higher probability of developing CEI and this was independent of CV and non-CV comorbidities regardless of inclusion/exclusion of pre-existing cardiac conditions. It is plausible that part of the higher burden of pre-existing CV comorbidities observed in the cases may reflect undiagnosed, pre-existing ATTR-CM. For instance, although coronary artery disease can coexist with ATTR-CM, elevated cardiac troponin levels—potentially resulting from amyloid-related myocardial injury^21^—may instead be attributed to acute coronary syndrome.

We demonstrate in a large U.S. population-based study that LSS—including surgically treated and medically managed—cases have a 2-fold higher rate of being diagnosed with amyloidosis and a 1.3-fold higher probability of developing CEI potentially related to undiagnosed ATTR-CM. It is notable that although the fold increase in CV events potentially indicating ATTR-CM is quite substantial, the absolute 10-year amyloidosis diagnosis rate was only 1.1%. Small studies have investigated the presence of ATTR deposits in the ligamentum flavum of patients undergoing LSS surgery, documenting that this finding is common;^7^, ^8^, ^9^, ^10^, ^11^ however, none^8^ to few^9^ patients have definite signs of ATTR-CM. In a prospective study from Belgium, 82 LSS surgery patients were screened by bone scintigraphy, monoclonal protein assessment, and spinal ligamentum flavum staining, and 59% had amyloid deposits in the ligamentum flavum; however, only 3 patients (3.7% of the whole population and 6.2% of the positive ligament patients) were diagnosed with ATTRwt-CM at the time of LSS surgery, all early stage and in individuals over 73 years old.^9^ Recently, a prospective follow-up study reported that around 15% of subjects (3/19 patients) with a high-degree ATTR deposition (classified as grade 3-4 deposition) in the ligamentous flavum at the time of LSS surgery were diagnosed with ATTRwt-CM 6 years later.^12^

We demonstrated that individuals with LSS had inferior survival as compared to the cases, but when patients were parsed by procedure code vs diagnosis code for LSS, LSS surgery cases had better survival than matched controls, likely a function of patient selection. Spinal surgery is an intermediate-risk surgery,^22^^,^^23^ and it is offered to more robust patients; indeed, patients undergoing LSS surgery in our cohort were younger and had fewer comorbidities at the time of the procedure.

For the CTS cohort, the results of our population-based study, which included patients with CTS who either did or did not undergo carpal tunnel release, are similar to those of a Danish Registry study,^18^ which included patients who underwent CTS release surgery. They reported that CTS patients were at a higher probability of developing CA, HF, atrial dysrhythmias, atrioventricular block, and PM/ICD implantation. Among their CTS patients, the HR and 10-year absolute cumulative incidence for a future amyloidosis diagnosis were 12.1 (95% CI: 4.37-33.6) and 0.1%, respectively, compared to our HR of 2.87 and cumulative 10-year incidence of 0.9%. Both studies demonstrated that despite more comorbidities among CTS patients than controls, there was no increase in mortality. The fact that mortality was no different between the CTS cases and controls could be due to low absolute rates of amyloidosis diagnoses, competing risk of death in elderly populations, or median follow-up times of only a decade. Regarding the differences in incident CEIs and overall survival between LSS and CTS cohort (Central Illustration), it is also worth considering that amyloid deposits, and therefore subsequent possible ATTR-CM, may be more prevalent in LSS than CTS specimens, as suggested by previous literature^7^^,^^8^^,^^10^^,^^14^^,^^24^

Although we did not seek histological or clinical confirmation in cases identified as incident diagnoses of amyloidosis as per ICD codes entered by treating physicians, previous literature reported that most histological amyloid deposits in patients with CTS and LSS is ATTR.^25^ Patients with a dual diagnosis of LSS and CTS had the highest probability of CV events (1.1- to 1.4-fold) and incident amyloidosis diagnosis (1.45%), underscoring that this subset of patients might warrant closer clinical evaluation. The notably higher incidence of HF and the other CEI, compared to the relatively low number of cases with an amyloid-related diagnosis, indicate a potential diagnostic gap, with a proportion of CEI cases potentially attributable to unrecognized CA. This observation, which needs to be contextualized in the study period when amyloidosis awareness was not as expanded as it is today, still highlights the need for increased clinical awareness in at-risk subsets.

Where do our and the existing published data leave us in terms of making recommendations about routinely sampling and staining these tissues at the time of surgery? The answer depends on the practitioner’s tolerance for positive tissue diagnoses with a relatively low absolute probability of ATTR-CM in the next decade. Early diagnosis in soft tissues may be of no clinical significance but potentially substantial emotional (and financial) burden. For the case of LSS surgery, the tissue is waste, and the Congo red stain is inexpensive, although typing does carry an additional cost. In contrast, in the case of CTS release, tissue is not always routinely taken, so the decision to remove tissue to screen for ATTR-CM may complicate a simple surgery.

### Study Limitations

There are several limitations to this study. First, this is an observational retrospective cohort study which makes assumptions, that is, the increased cardiac events observed in the cases as compared to the controls may be due to undiagnosed ATTR-CM; there is no biopsy proof that these assumptions are correct, although our results were consistent when adjusting for CV and non-CV comorbidities. Second, diagnoses, procedures, comorbidities, and outcomes were based on ICD codes as reported by treating physicians. Even though the predictive values of these codes have been reported before,^26^^,^^27^ undercoding or misdiagnosis based on this system may have occurred. Moreover, baseline comorbidities and outcomes were defined if at least 1 related ICD code was present, so a single coding was sufficient to establish a diagnosis or event. This improves sensitivity but it might decrease specificity (versus an approach requiring at least 2 codes on separate dates). Also, LSS/CTS surgery codes (which are likely more reliable than diagnosis codes due to reimbursement issues) were present only in a subset of patients. Third, the observation period of the study was during a time when ATTR-CM was likely underdiagnosed, thus affecting the analyses on incident amyloidosis diagnoses; however, we assume the relative risk should be accurate given the large sample size. Moreover, patients moving out of Olmsted County are lost to follow-up, a known limitation of REP data. However, since this affects both cases and controls similarly, it likely had minimal impact on our results. Finally, we did not look specifically at patients with bilateral CTS surgery, which might constitute an even higher-risk cohort, as this data are not currently available.

## Conclusions

In this U.S. population-based study, patients with LSS and CTS were at higher likelihood of CEI, had higher 10-year rates of amyloidosis diagnosis, and patients with LSS not undergoing surgery had the worst survival rates. These data highlight the importance of a dedicated assessment of CV symptoms for patients with LSS and/or CTS, particularly if both conditions and other red flags are present and regardless of surgical indications. Furthermore, they emphasize the need for cardiologists to actively investigate signs and symptoms of LSS and/or CTS in patients with clinical or imaging features suggestive of CA, as these may represent relevant diagnostic clues and early red flags. Although the presence of ATTR amyloid in ligaments may represent a “risk marker” for developing ATTR-CM, more information is required before recommending routine histological evaluation of these tissues, unless other risk factors co-exist.

Perspectives **COMPETENCY IN MEDICAL KNOWLEDGE:** Patients with LSS/CTS appear to be at a higher likelihood of cardiac events potentially related to undiagnosed ATTR-CM, with higher 10-year rates of amyloidosis diagnosis, and in some cases worst overall survival rates. However, the absolute rates of amyloidosis diagnoses remained low.

**TRANSLATIONAL OUTLOOK:** Further prospective studies are needed to address the relationship between CTS/LSS and the development of ATTR-CM and its timing. Moreover, future studies should focus on high-risk cohorts in which staining and analysis of CTS/LSS surgery specimens might be indicated.

## Funding support and author disclosures

Dr De Michieli has received honoraria from Pfizer Inc, Alnylam Pharmaceuticals, AstraZeneca Spa, and Euro Medica. Dr Dispenzieri has received research support from Alynlam, 10.13039/100004319Pfizer, Takeda, and BMS; she participates on the data and safety monitoring board for Oncopeptides and Sorrento; and is on the advisory board and independent review committee for Janssen. Dr Cipriani received honoraria from Pfizer Inc, Alnylam Pharmaceuticals, and AstraZeneca Spa. All other authors have reported that they have no relationships relevant to the contents of this paper to disclose.
