# Supplementary material for: Spinal Stenosis and Carpal Tunnel Syndrome as Surrogates of Transthyretin Amyloid Cardiomyopathy
Source: JACC Adv. 2026 Jan 20;5(2):102545. doi: 10.1016/j.jacadv.2025.102545 (PMC12856475; doi:10.1016/j.jacadv.2025.102545)
Supplement: Supplementary materials [file mmc1.pdf]

SPINAL STENOSIS AND CARPAL TUNNEL SYNDROME AS SURROGATES OF TRANSTHYRETIN AMYLOID CARDIOMYOPATHY

Laura De Michieli[1,2], Susan Geyer[3], Ellen McPhail[4], Mohamad Bydon[5], Benjamin D. Elder[5], Julie L. Rosenthal[6], Mary Jurisson[7], Sanjeev Kakar[8], Alberto Cipriani[2], Omar AbouEzzeddine[1], Surendra Dasari[9], Shaji Kumar[10], Morie Gertz[10], Martha Grogan[1], Angela Dispenzieri[10].

1. Department of Cardiovascular Medicine, Mayo Clinic and Medical School, Rochester, Minnesota, USA
2. Department of Cardiac, Thoracic and Vascular Sciences and Public Health, University of Padua, Padua, Italy
3. Department of Quantitative Health Sciences, Mayo Clinic, Rochester, MN.
4. Department of Laboratory Medicine and Pathology, Mayo Clinic, Rochester, Minnesota, USA
5. Department of Neurological Surgery, Mayo Clinic, Rochester, MN, USA
6. Department of Cardiovascular Medicine, Mayo Clinic, Phoenix, AZ
7. Department of Physical Medicine and Rehabilitation, Mayo Clinic, Rochester, MN
8. Division of Hand and Upper Extremity Surgery, Department of Orthopedics, Mayo Clinic, Rochester, MN.
9. Department of Qualitative Health Sciences, Mayo Clinic, Rochester, Minnesota, USA.
10. Division of Hematology, Mayo Clinic, Rochester, Minnesota, USA.

Supplemental material

Supplemental statistical methos.

Directed Acyclic Graphs (DAGs) reflecting the research questions for these matched analyses.

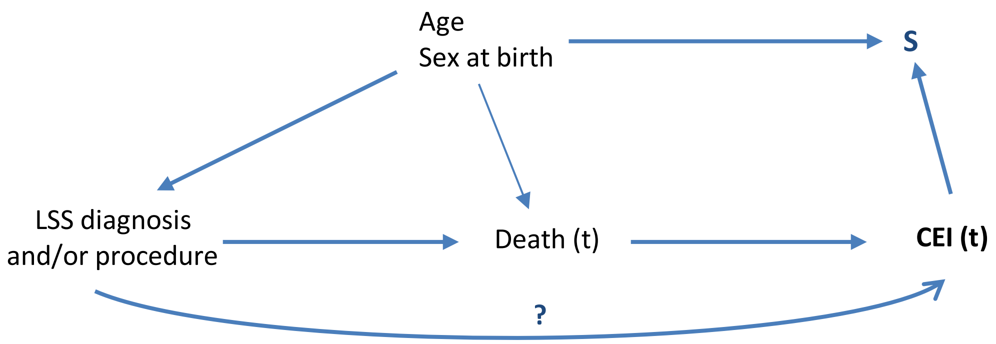

where **S** reflects the matched case-control sampling selection,
(t) reflects the event by time t,
CEI(t) is a cardiac event of interest by time t,
Death(t) is the competing event of death,

and $\xrightarrow{?}$ reflects the association of interest

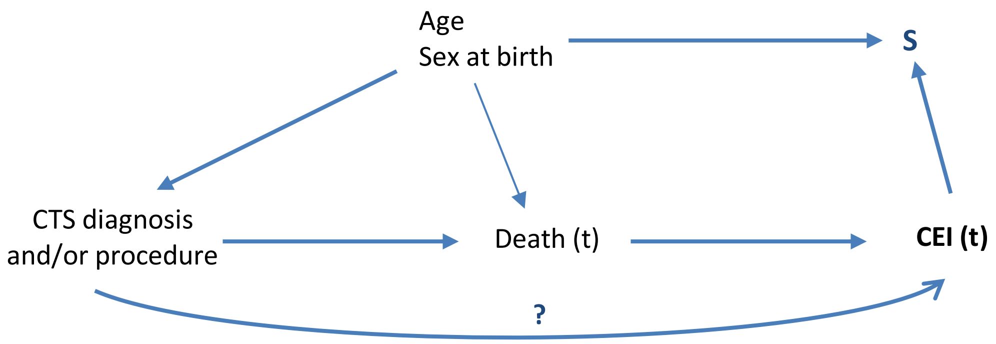

where **S** reflects the matched case-control sampling selection,
(t) reflects the event by time t,
CEI(t) is a cardiac event of interest by time t,
Death(t) is the competing event of death,

and $\xrightarrow{?}$ reflects the association of interest

Supplemental material

Supplemental Table 1. Diagnosis and procedure codes utilized for patient identification through the Rochester Epidemiology Project (REP) software.

	**Diagnosis codes**	**Procedure codes**
**Spinal stenosis diagnosis and surgery**	**ICD9**<br>724.02, 724.03 721.42, 756.12<br>**ICD10**<br>M43.06, M43.16, M48.061, M48.062, M47.16, M48.07	**CPT**<br>00NY0ZZ, 00NY3ZZ, 00NY4ZZ<br>63005, 63012, 63017, 63042, 63044, 63047, 63048.<br>**ICD 9**<br>3.02, 03.09, 81.00, 81.30, 81.34-81.38, 81.04-81.08
**Carpal tunnel diagnosis and surgery**	**ICD9**<br>354.0<br>**ICD10**<br>G56.00, G56.01, G56.02, G56.03	**CPT**<br>64721, 29848, 04.43

Supplemental Table 2. Diagnosis codes utilized for outcomes and amyloidosis diagnosis identification through the Rochester Epidemiology Project (REP) software.

**Cardiovascular outcomes**	**ICD10**	**Cardiovascular outcomes**	**ICD9**
Heart failure	I50	Heart failure	428.
Left heart failure	I50.1	Congestive heart failure, unspecified	428.0
Systolic heart failure	I50.20, I50.21, I50.22, I50.23	Left heart failure	428.1
Diastolic heart failure	I50.30, I50.31, I50.32, I50.33	Systolic heart failure	428.2
Combined heart failure	I50.40, I50.41, I50.42, I50.43	Diastolic heart failure	428.3
Heart failure, unspecified	I50.83, I50.84, I50.89, I50.9	Combined heart failure	428.4
Right heart failure, unspecified	I50.810, I50.811, I50.812, I50.813, I50.84	Heart failure, unspecified	428.9
Biventricular heart failure	I50.82	Atrial fibrillation and flutter	427.3
Atrial Fibrillation	I4.80, I4.811, I4.819, I4.820, I4.821,	Atrial fibrillation	427.31
Atrial flutter	I4.891<br>I4.83, I4.84, I4.892	Atrial flutter	427.32
**Amyloidosis Diagnosis**	**ICD10**	**Amyloidosis Diagnosis**	**ICD9**
Non-neuropathic heredofamilial amyloidosis	E85.0	Amyloidosis	277.3
Neuropathic heredofamilial amyloidosis	E85.1	Amyloidosis, unspecifed	277.30
Heredofamilial amyloidosis, unspecified	E85.2	Familial mediterranean fever	277.31
Secondary systemic amyloidosis	E85.3	Other amyloidosis	277.39
Organ-limited amyloidosis	E85.4		
Other amyloidosis	E85.8		
Light chain (AL) amyloidosis	E85.81		
Wild-type transthyretin-related (ATTR) amyloidosis	E85.82		
Other amyloidosis	E85.89		
Amyloidosis, unspecified	E85.9		

Supplemental Table 3. Baseline clinical characteristics of patients with both LSS and CTS, compared to those of patients with a single diagnosis and controls.

Characteristic	CTS only cases N=5,488	LSS only cases N=4,902	LSS+CTS cases N=1,181	Matched-controls for LSS+CTS cases N=3,219	p-value for LSS+CTS cases vs CTS only cases	p-value for LSS+CTS cases vs LSS only cases	p-value for LSS+CTS cases vs. matched-controls**
Sex F, n (%)	3,578 (65.2)	2,597 (53.0)	753 (63.8)	2,037 (63.3)	0.37	<0.0001	
Age at index date, mean (SD)	62.4 (10.6)	68.8 (10.9)	66.6 (10.6)	62.4 (11.1)	<0.0001	<0.0001	
**Prior history of:**							
Heart failure, n (%)	302 (5.5)	523 (10.7)	156 (13.2)	132 (4.1)	<0.0001	0.015	<0.0001
Atrial fib/flutter, n (%)	1,380 (25.1)	565 (11.5)	331 (28.0)	388 (12.1)	0.043	<0.0001	<0.0001
PM/ICD, n (%)	110 (2.0)	167 (3.4)	33 (2.8)	47 (1.5)	0.11	0.33	0.65
HF, AF/AFL, and/or PM/ICD, n (%)	1,484 (27.0)	878 (17.9)	366 (31.0)	459 (14.3)	0.007	<0.0001	<0.0001
Systemic hypertension, n (%)	2,897 (52.8)	3,322 (67.8)	762 (64.5)	1,277 (40.0)	<0.0001	0.036	<0.0001
Diabetes mellitus, n (%)	1,923 (35.0)	2,066 (42.1)	466 (39.5)	670 (20.8)	0.005	0.10	<0.0001
Hyperlipidemia, n (%)	3,428 (62.5)	3,485 (71.1)	826 (69.9)	1,491 (46.3)	<0.0001	0.46	<0.0001
Coronary artery disease, n (%)	1,014 (18.5)	1,456 (29.7)	342 (29.0)	444 (13.8)	<0.0001	0.64	<0.0001
Previous MI, n (%)	290 (5.3)	454 (9.3)	87 (7.4)	128 (4.0)	0.006	0.046	0.002
Cerebrovascular disease, n (%)	546 (9.9)	990 (20.2)	169 (14.3)	235 (7.3)	<0.0001	<0.0001	0.002
Peripheral vascular disease, n (%)	814 (14.8)	1,438 (29.3)	264 (22.4)	268 (8.3)	<0.0001	<0.0001	<0.0001
Chronic kidney disease, n (%)	382 (7.0)	576 (11.8)	94 (8.0)	123 (3.8)	0.25	0.0002	0.003
COPD, n (%)	506 (9.2)	721 (14.7)	140 (11.9)	220 (6.8)	0.006	0.013	0.0004
Malignancy (any type excluding skin tumors), n (%)	1,180 (21.5)	1,635 (33.4)	317 (26.8)	596 (18.5)	<0.0001	<0.0001	0.009
Number of comorbidities at baseline*, mean (SD)	2.4 (1.9)	3.3 (2.1)	2.9 (2.0)	1.7 (1.8)	<0.0001	<0.0001	<0.0001

*Comorbidities at baseline (i.e. prior to and up to 90 days after the index date), not including HF, AF/AFL, and/or PM/ICD

**P-values based on conditional logistic regression that accommodate cases and matched-controls

Abbreviations: LSS: lumbar spinal stenosis; SD: standard deviation; Atrial fib: atrial fibrillation; PM: pacemaker; ICD: implantable cardiac defibrillator; COPD: chronic obstructive pulmonary disease; NA: not applicable.

Supplemental Table 4: Model results for competing risk regression models for LSS cases/CTS cases versus their matched-controls, regardless of previous history of the cardiac event of interest. All models evaluate the cumulative incidence of the cardiac event of interest with a competing risk of death, and all models adjust for age. Table values reflect the cause-specific hazard ratio (csHR) of the case or type of case in relation to the age- and sex-matched-controls, as well as the corresponding 95% confidence interval for the csHR. Abbreviations: AF/AFL, atrial fibrillation/flutter; CHF, congestive heart failure; CTS, carpal tunnel syndrome; LSS, lumbar spinal stenosis; PM/ICD, pacemaker/implantable cardioverter defibrillator.

	**Cardiac event of interest**				
**Models**	**CHF**	**AF/AFL**	**PM/ICD**	**Any of these cardiac events (CHF, AF/AFL, and/or cardiac device)**	**Corresponding figures**
LSS cohort					
LSS case (vs. control)	1.38 (1.36 – 1.41)	1.20 (1.18 – 1.21)	NA	1.15 (1.13 – 1.16),	Suppl. 2A, 2B, 2C
CTS cohort					
CTS case (vs. control)	1.35 (1.33 – 1.38)	1.25 (1.23 – 1.26)	NA	1.17 (1.15 – 1.18)	Suppl. 2D, 2E, 2F

Supplemental Table 5. Sex-based differences in the cardiac events of interest. Abbreviations: AF/AFL, atrial fibrillation/flutter; CHF, congestive heart failure; CTS, carpal tunnel syndrome; LSS, lumbar spinal stenosis; PM/ICD, pacemaker/implantable cardioverter defibrillator.

	**LSS**			**CTS**		
	**Women**	**Men**	**p-value**	**Women**	**Men**	**p-value**
CHF						
All patients						
5-year rate	8.3%	8.9%	0.002	5.4%	7.1%	<0.0001
10-year rate	15.4%	17.4%		10.9%	13.9%	
Cases only						
5-year rate	11.3%	11.6%	0.82	7.0%	8.5%	0.0003
10-year rate	22.3%	22.7%		14.3%	17.5%	
Controls for cases						
5-year rate	7.2%	8.0%	0.0002	4.8%	6.5%	<0.0001
10-year rate	12.9%	15.4%		9.6%	12.6%	
AF/flutter						
All patients						
5-year rate	20.2%	24.1%	<0.0001	13.2%	17.2%	<0.0001
10-year rate	35.0%	39.8%		24.9%	31.4%	
Cases only						
5-year rate	26.0%	28.7%	0.010	15.5%	20.7%	<0.0001
10-year rate	43.4%	45.6%		30.6%	38.1%	
Controls for cases						
5-year rate	18.1%	22.4%	<0.0001	12.4%	16.0%	<0.0001
10-year rate	32.0%	37.8%		22.9%	29.1%	
PM/ICD						
All patients						
5-year rate	1.6%	2.8%	<0.0001	1.2%	1.6%	<0.0001
10-year rate	3.0%	5.1%		2.3%	3.7%	
Cases only						
5-year rate	2.0%	3.9%	<0.0001	1.3%	1.9%	<0.0001
10-year rate	4.2%	7.2%		3.0%	4.8%	
Controls for cases						
5-year rate	1.4%	2.3%	<0.0001	1.1%	1.4%	<0.0001
10-year rate	2.6%	4.3%		2.1%	3.3%	
Any cardiac event of interest						
All patients						
5-year rate	12.4%	15.0%	<0.0001	7.5%	9.6%	<0.0001
10-year rate	27.5%	31.8%		17.6%	23.5%	
Cases only						
5-year rate	17.0%	18.6%	0.17	9.0%	12.2%	<0.0001
10-year rate	36.1%	36.5%		22.0%	29.0%	
Controls for cases						
5-year rate	10.7%	13.8%	<0.0001	6.9%	8.7%	<0.0001
10-year rate	24.5%	30.1%		16.0%	21.7%	

Supplemental Table 6. Risk of cardiac events of interest in the cohort of patients with both LSS and CTS. Patients with a prior history of the cardiac event of interest are excluded. Abbreviations: AF/AFL, atrial fibrillation/flutter; CHF, congestive heart failure; CTS, carpal tunnel syndrome; LSS, lumbar spinal stenosis; PM/ICD, pacemaker/implantable cardioverter defibrillator.

Group	N*	csHR	95% CI	p-value	Est. 10-year rate (95% CI)	Est. 20-year rate (95% CI)
CHF (suppl. Fig 9 A/B)						
Controls	3087	ref			11.7% (10.5 – 13.0%)	23.0% (21.0 – 25.2%)
LSS+CTS cases overall	1101	1.95	1.71 – 2.22	<0.0001	19.3% (16.7 – 21.9%)	39.8% (35.7 – 43.8%)
LSS+CTS within a year of each other	179	2.48	1.76 – 3.49	<0.0001	28.1% (20.5 – 36.2%)	40.0% (28.1 – 51.6%)
CTS first, then LSS	560	1.56	1.30 – 1.88	<0.0001	11.6% (9.0 – 14.5%)	31.7% (26.8 – 36.7%)
LSS first, then CTS	362	2.40	1.92 – 2.99	<0.0001	31.3% (25.3 – 37.5%)	58.2% (49.6 – 65.8%)
AF/AFL (suppl. Fig 10 A/B)						
Controls	2831	ref			30.2% (28.3 – 32.0%)	46.6% (43.9 – 49.3%)
LSS+CTS cases overall	850	1.52	1.36 – 1.70	<0.0001	43.0% (39.3 – 46.6%)	66.7% (62.1 – 70.8%)
LSS+CTS within a year of each other	142	1.51	1.13 – 2.01	0.005	52.4% (42.0 – 61.8%)	64.6% (51.0 – 75.3%)
CTS first, then LSS	462	1.65	1.42 – 1.92	<0.0001	38.3% (33.7 – 42.9%)	63.6% (57.5 – 69.0%)
LSS first, then CTS	246	1.32	1.08 – 1.62	0.007	49.4% (41.5 – 56.7%)	74.0% (65.3 – 80.9%)
PM/ICD implantation (Suppl. Fig 11 A/B)						
Controls	3172	ref			2.8% (2.3 – 3.5%)	5.3% (4.3 – 6.5%)
LSS+CTS cases overall	1148	1.72	1.33 – 2.23	<0.0001	4.1% (3.0 – 5.6%)	10.1% (7.8 – 12.7%)
LSS+CTS within a year of each other	195	1.59	0.84 – 3.03	0.16	5.1% (2.2 – 9.9%)	13.8% (6.2 – 24.3%)
CTS first, then LSS	572	1.76	1.21 – 2.55	0.003	2.3% (1.2 – 3.8%)	8.0% (5.4 – 11.2%)
LSS first, then CTS	381	1.85	1.22 – 2.80	0.004	7.7% (4.7 – 11.6%)	13.5% (8.9 – 19.1%)
Combined cardiac endpoint (suppl. Fig 12 A/B)						
Controls	2760	ref			21.0% (19.3 – 22.7%)	50.4% (47.7 – 53.1%)

LSS+CTS cases overall	815	1.61	1.44 – 1.81	<0.0001	29.7% (26.3 – 33.2%)	65.6% (61.1 – 69.7%)
LSS+CTS within a year of each other	133	1.57	1.16 – 2.13	0.004	41.0% (31.1 – 50.7%)	76.2% (60.7 – 86.3%)
CTS first, then LSS	453	1.51	1.29 – 1.77	<0.0001	21.7% (17.9 – 25.8%)	58.3% (52.4 – 63.7%)
LSS first, then CTS	229	1.82	1.48 – 2.24	<0.0001	43.8% (35.8 – 51.4%)	78.8% (70.4 – 85.1%)

Supplemental Figures Legend

Supplemental Figure 1. Consort diagrams for lumbar spinal stenosis (LSS, panel A) and carpal tunnel syndrome (CTS, panel B) cohorts.

Supplemental Figure 2. Cumulative incidence of CHF, AF/AFL, PM/ICD implantation and of the composite outcome in patients with LSS (Panel A-D) and CTS (panel E-H), with and without a procedure performed, and matched-controls in those without a prior history of the cardiac event of interest. Abbreviations: AF/AFL, atrial fibrillation/flutter; CHF, congestive heart failure; csHR, cause specific hazard ratio; CTS, carpal tunnel syndrome; LSS, lumbar spinal stenosis; PM/ICD, pacemaker/implantable cardioverter defibrillator.

Supplemental Figure 3. Cumulative incidence of cardiac events in patients with lumbar spinal stenosis (LSS, panel A-C) and carpal tunnel syndrome (CTS, panel D-F) regardless of previous history of each of the cardiac events of interest. Abbreviations as per Supplemental Figure 2.

Supplemental Figure 4. Forest plot with multivariable competing risk regression model for any cardiac event of interest (HF, AF/AFL, PM/ICD implantation) in the LSS cohort (panel A) and CTS cohort (Panel B). Abbreviations: CAD, coronary artery disease; COPD, chronic obstructive pulmonary disease; CKD, chronic kidney disease. Other abbreviations as per Supplemental Figure 2.

Supplemental Figure 5. Cumulative incidence of cardiac events of interest in cases with LSS and CTS (both diagnoses) vs matched controls, excluding patients with a prior history of the cardiac event of interest. Panel A-D, overall incidence in cases vs controls. Panel E-H, patients are categorized accordingly to the timing of the two diagnoses (LSS and CTS). Abbreviations as per Supplemental Figure 2.

Supplemental Figure 6 Cumulative incidence of amyloidosis across patients with LSS versus matched controls (Panel A) and patients with CTS versus matched controls (Panel B), where death without amyloidosis is treated as a competing risk (cumulative incidence of death is also reported). Abbreviations as per Supplemental Figure 2.

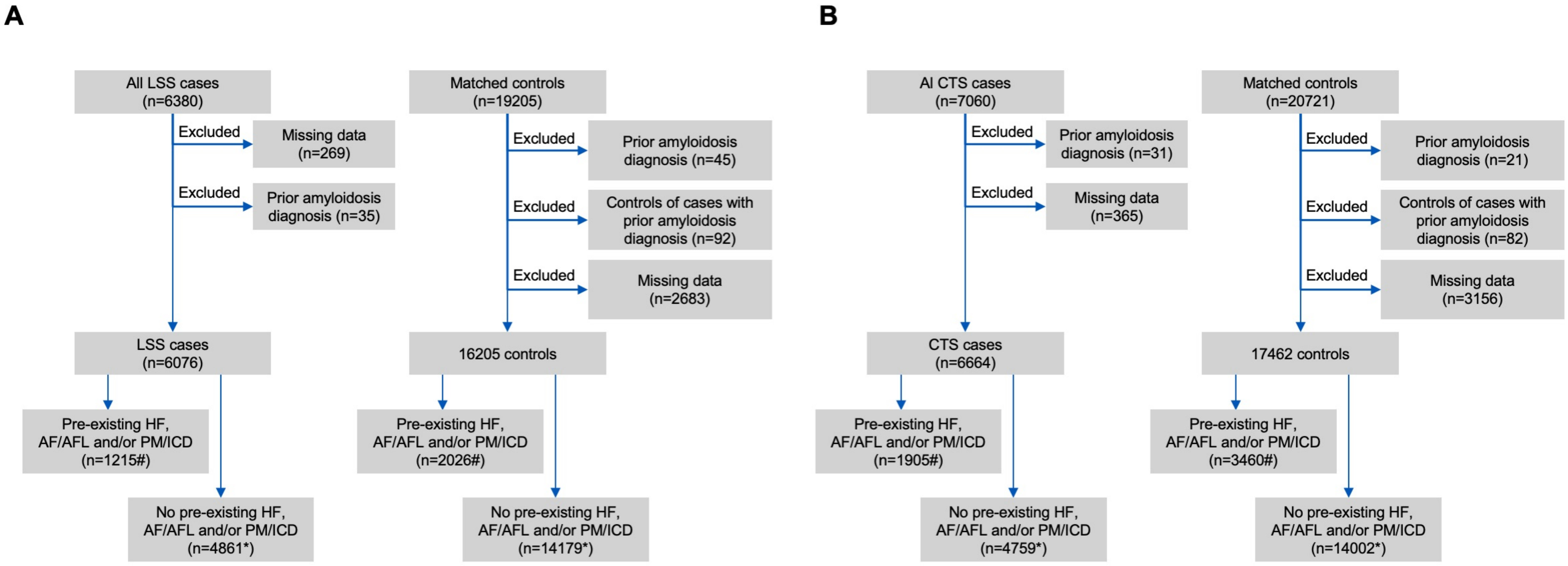

Supplemental Figure 1

Suppl. Figure 2

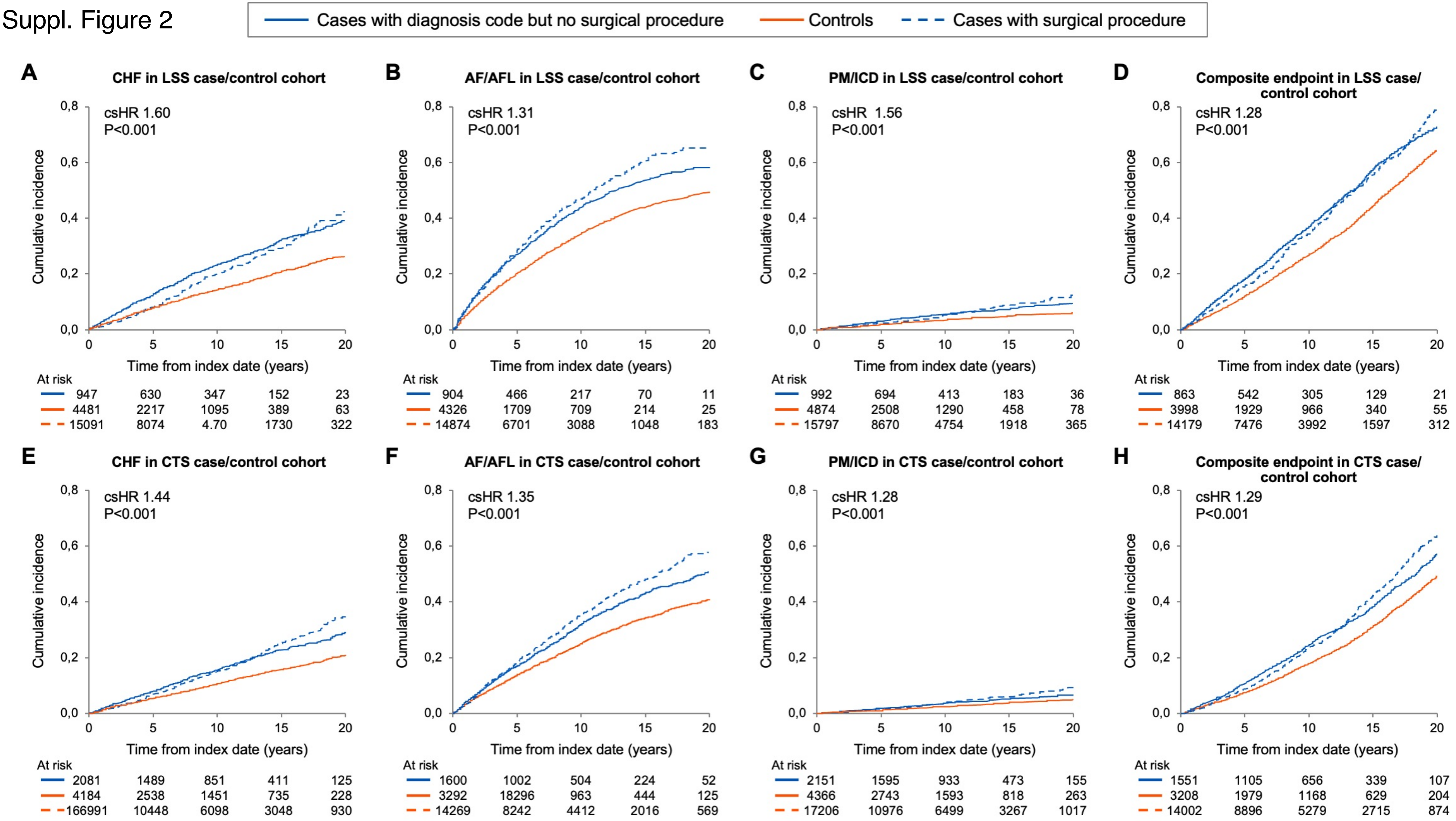

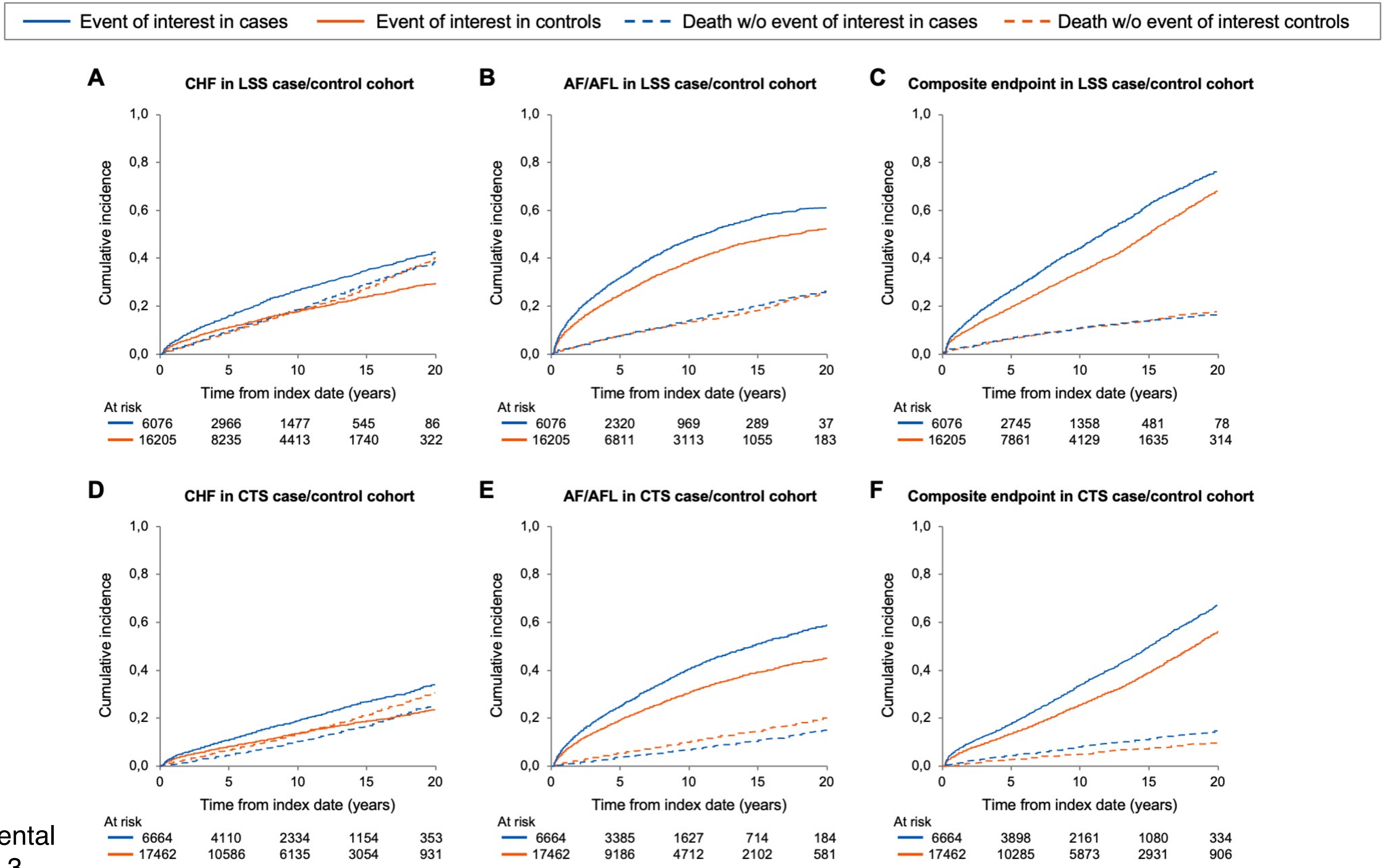

Supplemental Figure 3

©2025 Mayo Foundation for Medical Education and Research | WF5524462-6

A

Multivariable model results for cumulative incidence of HF, AF/AFL, and/or PMI/ICD in LSS cases and matched controls

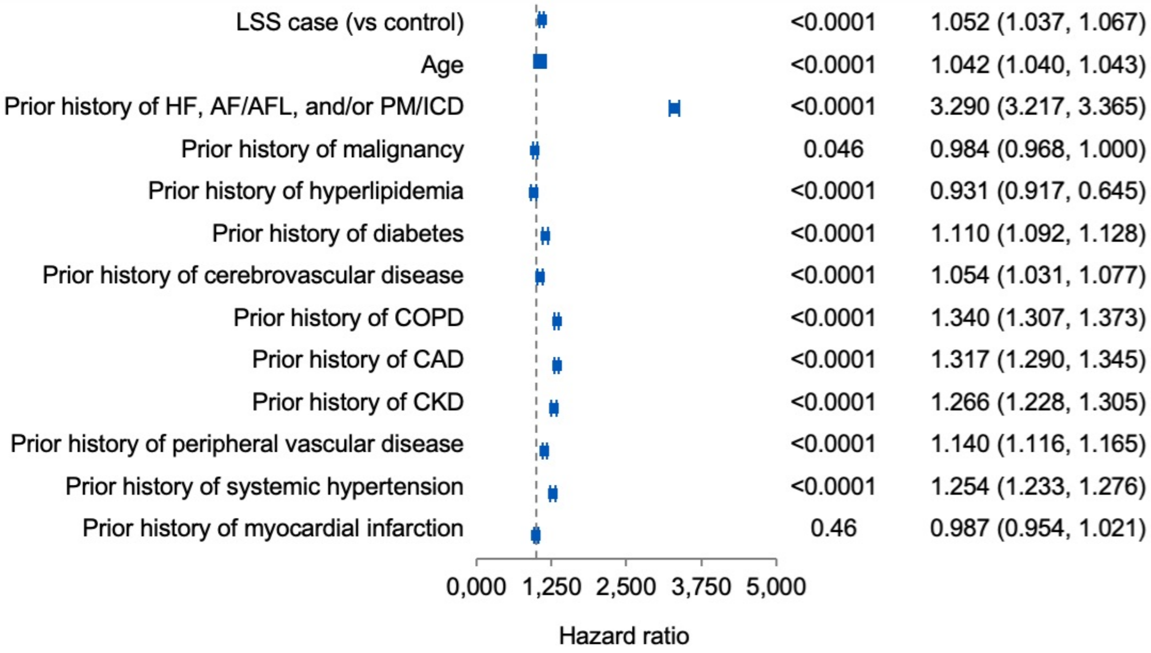

	P	HR (95% C)
LSS case (vs control)	<0.0001	1.052 (1.037, 1.067)
Age	<0.0001	1.042 (1.040, 1.043)
Prior history of HF, AF/AFL, and/or PM/ICD	<0.0001	3.290 (3.217, 3.365)
Prior history of malignancy	0.046	0.984 (0.968, 1.000)
Prior history of hyperlipidemia	<0.0001	0.931 (0.917, 0.645)
Prior history of diabetes	<0.0001	1.110 (1.092, 1.128)
Prior history of cerebrovascular disease	<0.0001	1.054 (1.031, 1.077)
Prior history of COPD	<0.0001	1.340 (1.307, 1.373)
Prior history of CAD	<0.0001	1.317 (1.290, 1.345)
Prior history of CKD	<0.0001	1.266 (1.228, 1.305)
Prior history of peripheral vascular disease	<0.0001	1.140 (1.116, 1.165)
Prior history of systemic hypertension	<0.0001	1.254 (1.233, 1.276)
Prior history of myocardial infarction	0.46	0.987 (0.954, 1.021)

B

Multivariable model results for cumulative incidence of HF, AF/AFL, and/or PMI/ICD in CTS cases and matched controls

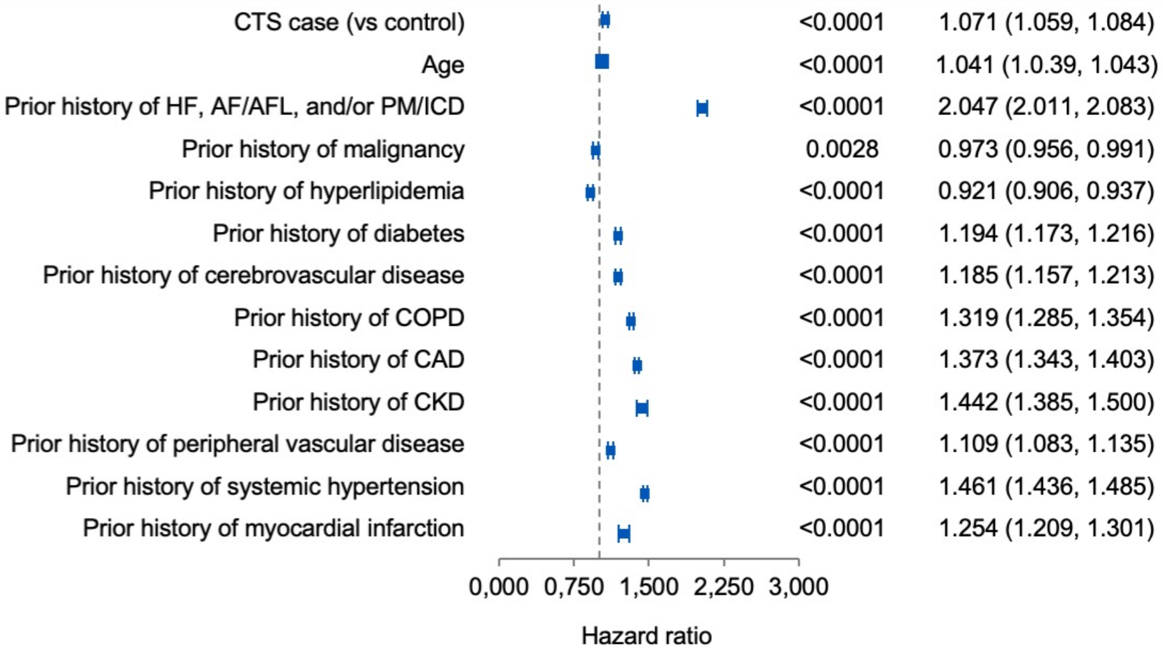

	P	HR (95% C)
CTS case (vs control)	<0.0001	1.071 (1.059, 1.084)
Age	<0.0001	1.041 (1.0.39, 1.043)
Prior history of HF, AF/AFL, and/or PM/ICD	<0.0001	2.047 (2.011, 2.083)
Prior history of malignancy	0.0028	0.973 (0.956, 0.991)
Prior history of hyperlipidemia	<0.0001	0.921 (0.906, 0.937)
Prior history of diabetes	<0.0001	1.194 (1.173, 1.216)
Prior history of cerebrovascular disease	<0.0001	1.185 (1.157, 1.213)
Prior history of COPD	<0.0001	1.319 (1.285, 1.354)
Prior history of CAD	<0.0001	1.373 (1.343, 1.403)
Prior history of CKD	<0.0001	1.442 (1.385, 1.500)
Prior history of peripheral vascular disease	<0.0001	1.109 (1.083, 1.135)
Prior history of systemic hypertension	<0.0001	1.461 (1.436, 1.485)
Prior history of myocardial infarction	<0.0001	1.254 (1.209, 1.301)

Supplemental Figure 4

Suppl. Figure 5

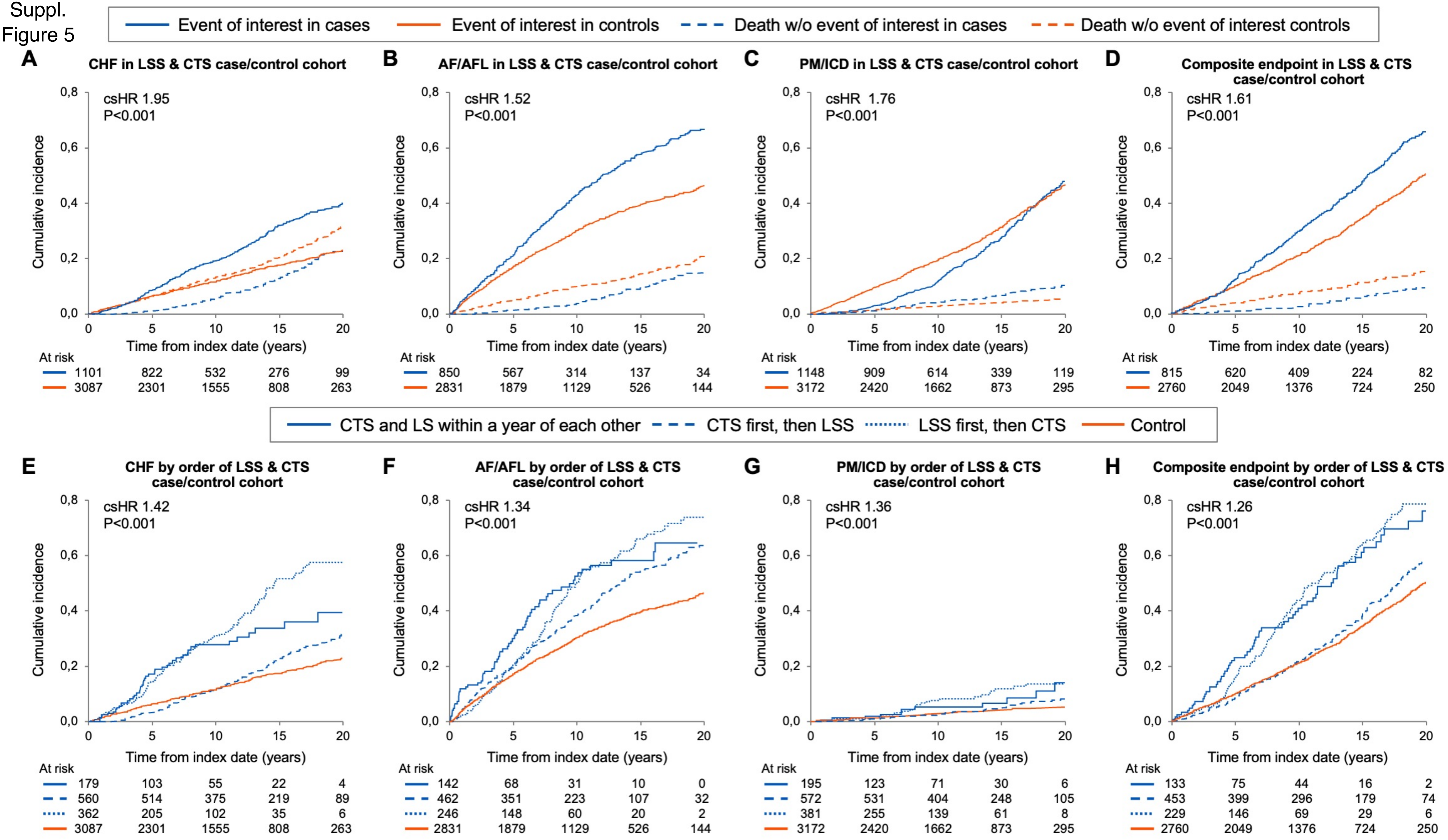

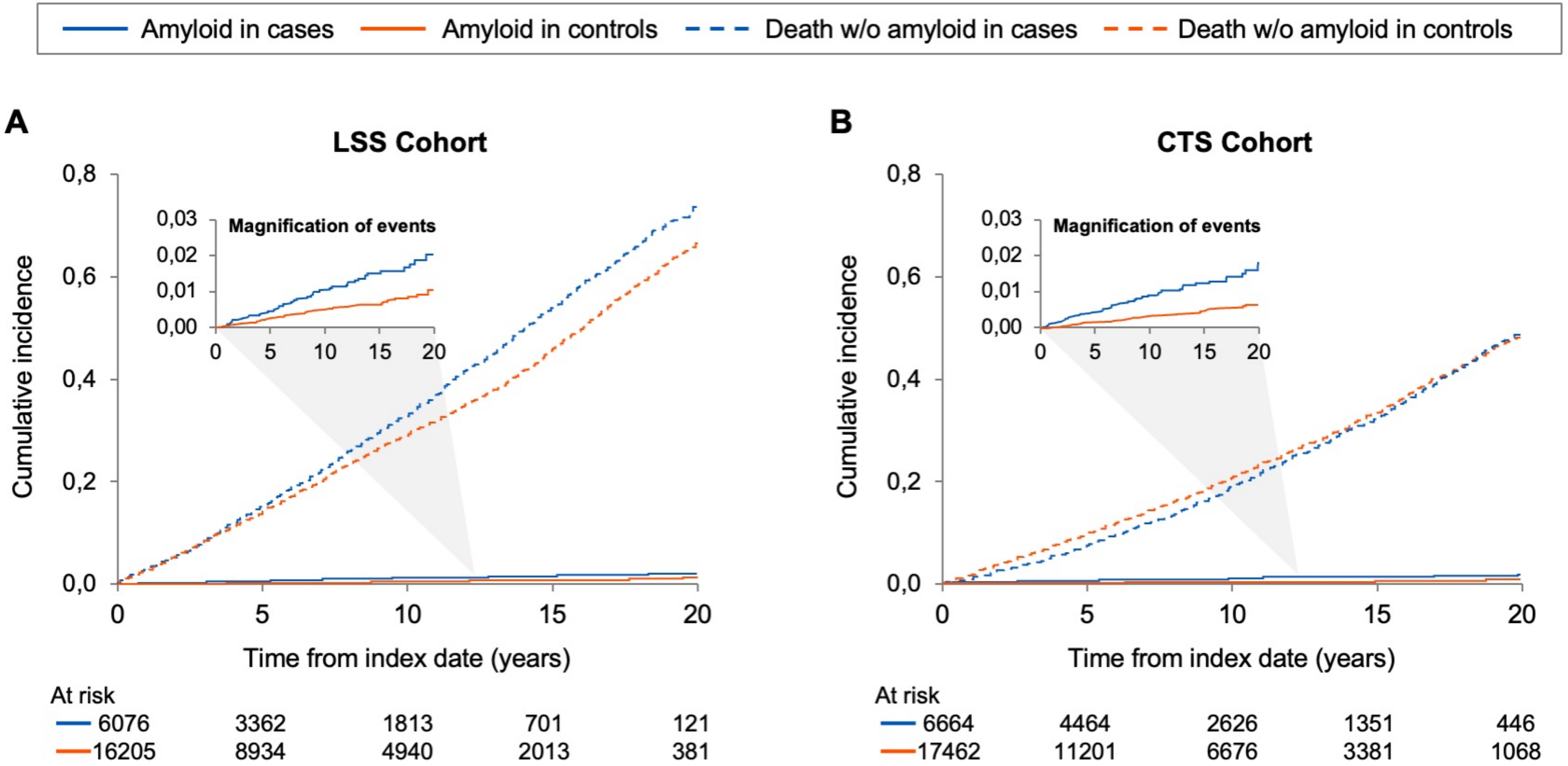

Supplemental Figure 6

STROBE Statement—checklist of items that should be included in reports of observational studies

	Item No.	Recommendation	Page No.	Relevant text from manuscript
**Title and abstract**	1	(*a*) Indicate the study's design with a commonly used term in the title or the abstract	3	Retrospective population-based cohort study
		(*b*) Provide in the abstract an informative and balanced summary of what was done and what was found	3	See abstract
**Introduction**				
Background/rationale	2	Explain the scientific background and rationale for the investigation being reported	5	
Objectives	3	State specific objectives, including any prespecified hypotheses	5-6	
**Methods**				
Study design	4	Present key elements of study design early in the paper	6	
Setting	5	Describe the setting, locations, and relevant dates, including periods of recruitment, exposure, follow-up, and data collection	6	Retrospective cohort study of patients between 50 and 90 years old with a history of LSS and/or CTS documented between 1995 and 2015, identified among residents of Olmsted County (Minnesota, USA)
Participants	6	(*a*) *Cohort study*—Give the eligibility criteria, and the sources and methods of selection of participants. Describe methods of follow-up<br>*Case-control study*—Give the eligibility criteria, and the sources and methods of case ascertainment and control selection. Give the rationale for the choice of cases and controls<br>*Cross-sectional study*—Give the eligibility criteria, and the sources and methods of selection of participants	6-7	
		(*b*) *Cohort study*—For matched studies, give matching criteria and number of exposed and unexposed<br>*Case-control study*—For matched studies, give matching criteria and the number of controls per case	6-7	

Variables	7	Clearly define all outcomes, exposures, predictors, potential confounders, and effect modifiers. Give diagnostic criteria, if applicable	7
Data sources/ measurement	8*	For each variable of interest, give sources of data and details of methods of assessment (measurement). Describe comparability of assessment methods if there is more than one group	7-8
Bias	9	Describe any efforts to address potential sources of bias	7-8
Study size	10	Explain how the study size was arrived at	7-8

Continued on next page

Quantitative variables	11	Explain how quantitative variables were handled in the analyses. If applicable, describe which groupings were chosen and why	8
Statistical methods	12	(*a*) Describe all statistical methods, including those used to control for confounding	8
		(*b*) Describe any methods used to examine subgroups and interactions	8
		(*c*) Explain how missing data were addressed	8
		(*d*) *Cohort study*—If applicable, explain how loss to follow-up was addressed<br>*Case-control study*—If applicable, explain how matching of cases and controls was addressed<br>*Cross-sectional study*—If applicable, describe analytical methods taking account of sampling strategy	8
		(*e*) Describe any sensitivity analyses	8
**Results**			
Participants	13*	(a) Report numbers of individuals at each stage of study—eg numbers potentially eligible, examined for eligibility, confirmed eligible, included in the study, completing follow-up, and analysed	8-9
		(b) Give reasons for non-participation at each stage	8-9
		(c) Consider use of a flow diagram	Supplemental Figure 1
Descriptive data	14*	(a) Give characteristics of study participants (eg demographic, clinical, social) and information on exposures and potential confounders	8-9
		(b) Indicate number of participants with missing data for each variable of interest	Supplemental Figure 1
		(c) *Cohort study*—Summarise follow-up time (eg, average and total amount)	9-13
Outcome data	15*	*Cohort study*—Report numbers of outcome events or summary measures over time	9-13
		Case-control study—Report numbers in each exposure category, or summary measures of exposure	
		Cross-sectional study—Report numbers of outcome events or summary measures	
Main results	16	(*a*) Give unadjusted estimates and, if applicable, confounder-adjusted estimates and their precision (eg, 95% confidence interval). Make clear which confounders were adjusted for and why they were included	9-13
		(*b*) Report category boundaries when continuous variables were categorized	9-13
		(*c*) If relevant, consider translating estimates of relative risk into absolute risk for a meaningful time period	9-13

Continued on next page

Other analyses	17	Report other analyses done—eg analyses of subgroups and interactions, and sensitivity analyses	9-13
**Discussion**			
Key results	18	Summarise key results with reference to study objectives	13
Limitations	19	Discuss limitations of the study, taking into account sources of potential bias or imprecision. Discuss both direction and magnitude of any potential bias	17
Interpretation	20	Give a cautious overall interpretation of results considering objectives, limitations, multiplicity of analyses, results from similar studies, and other relevant evidence	14-16
Generalisability	21	Discuss the generalisability (external validity) of the study results	14-16
**Other information**			
Funding	22	Give the source of funding and the role of the funders for the present study and, if applicable, for the original study on which the present article is based	2

*Give information separately for cases and controls in case-control studies and, if applicable, for exposed and unexposed groups in cohort and cross-sectional studies.

Note: An Explanation and Elaboration article discusses each checklist item and gives methodological background and published examples of transparent reporting. The STROBE checklist is best used in conjunction with this article (freely available on the Web sites of PLoS Medicine at http://www.plosmedicine.org/, Annals of Internal Medicine at http://www.annals.org/, and Epidemiology at http://www.epidem.com/). Information on the STROBE Initiative is available at www.strobe-statement.org.
